# Corrigendum: Modeling DNA Opening in the Eukaryotic Transcription Initiation Complexes *via* Coarse-Grained Models

**DOI:** 10.3389/fmolb.2021.817343

**Published:** 2021-12-07

**Authors:** Genki Shino, Shoji Takada

**Affiliations:** Department of Biophysics, Graduate School of Science, Kyoto University, Kyoto, Japan

**Keywords:** transcription, eukaryotes, protein-DNA complex, DNA opening, molecular dynamics simulation

In the original article, we did not include the **Funding Information**. “This work was supported by the Ministry of Education, Culture, Sport, Science, and Technology (MEXT) grant JPMXP1020200101 as “Program for Promoting Researches on the Supercomputer Fugaku” (ST), by the Japan Science and Technology Agency (JST) grant (JPMJCR1762) (ST), and by Japan Society for the Promotion of Science (Award number(s): 20H05934, 21H02441).”

The authors apologize for this error and state that this does not change the scientific conclusions of the article in any way. The original article has been updated.

