# Supplementary figures and images for: Modeling DNA Opening in the Eukaryotic Transcription Initiation Complexes via Coarse-Grained Models
Source: Front Mol Biosci. 2021 Nov 15;8:772486. doi: 10.3389/fmolb.2021.772486 (PMC8636136; doi:10.3389/fmolb.2021.772486)

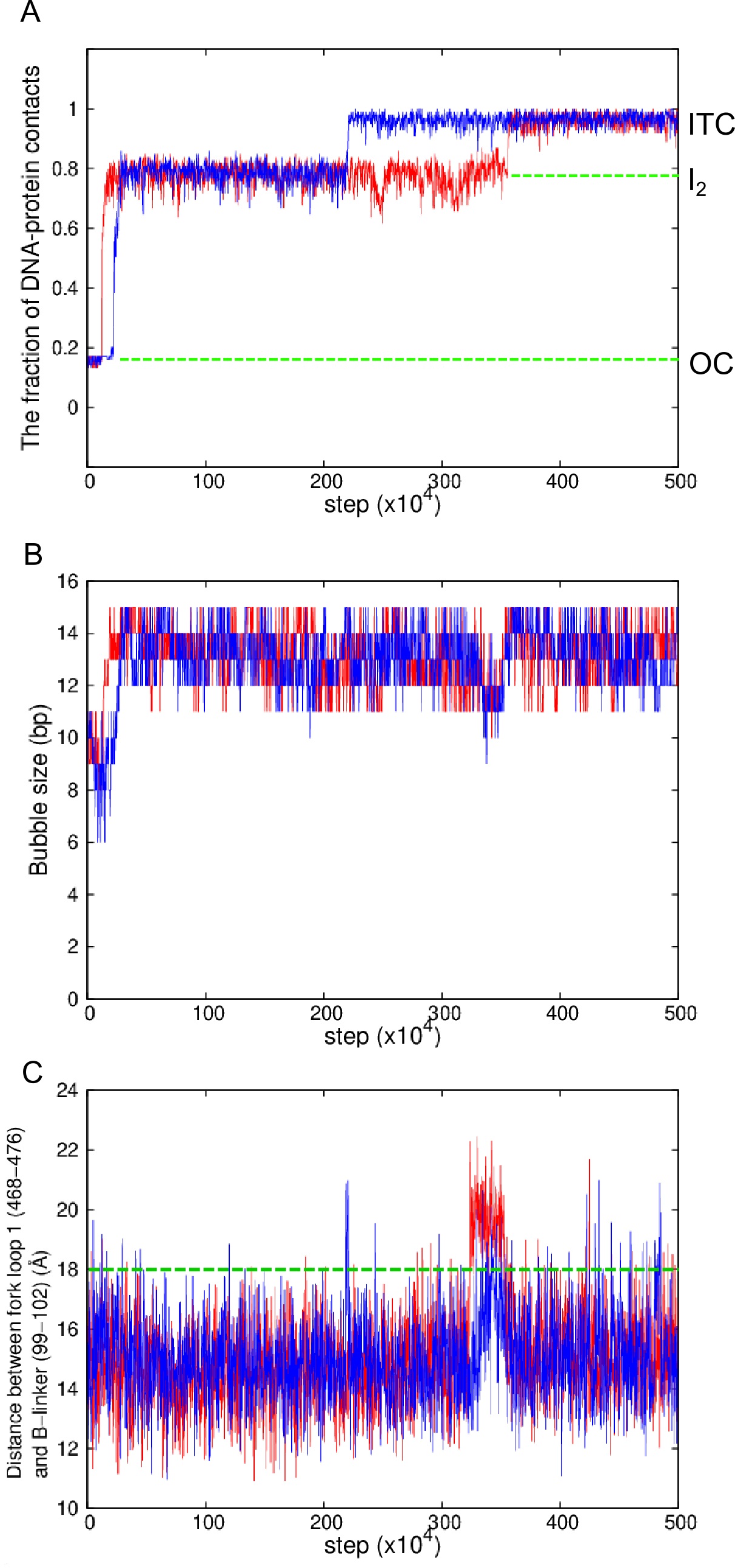

Supplement: Supplementary file 1 [file Image3.JPEG]

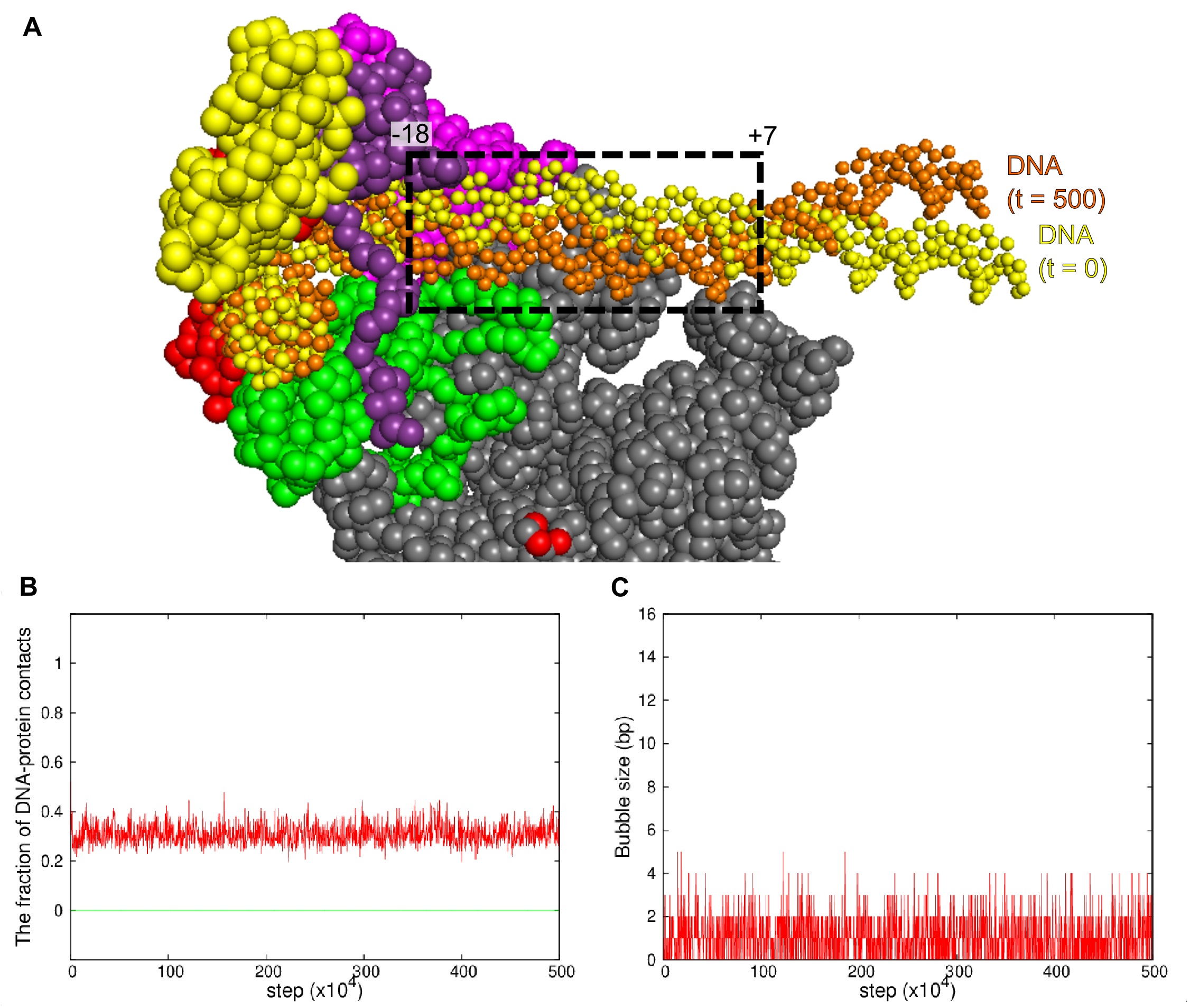

Supplement: Supplementary file 2 [file Image1.JPEG]

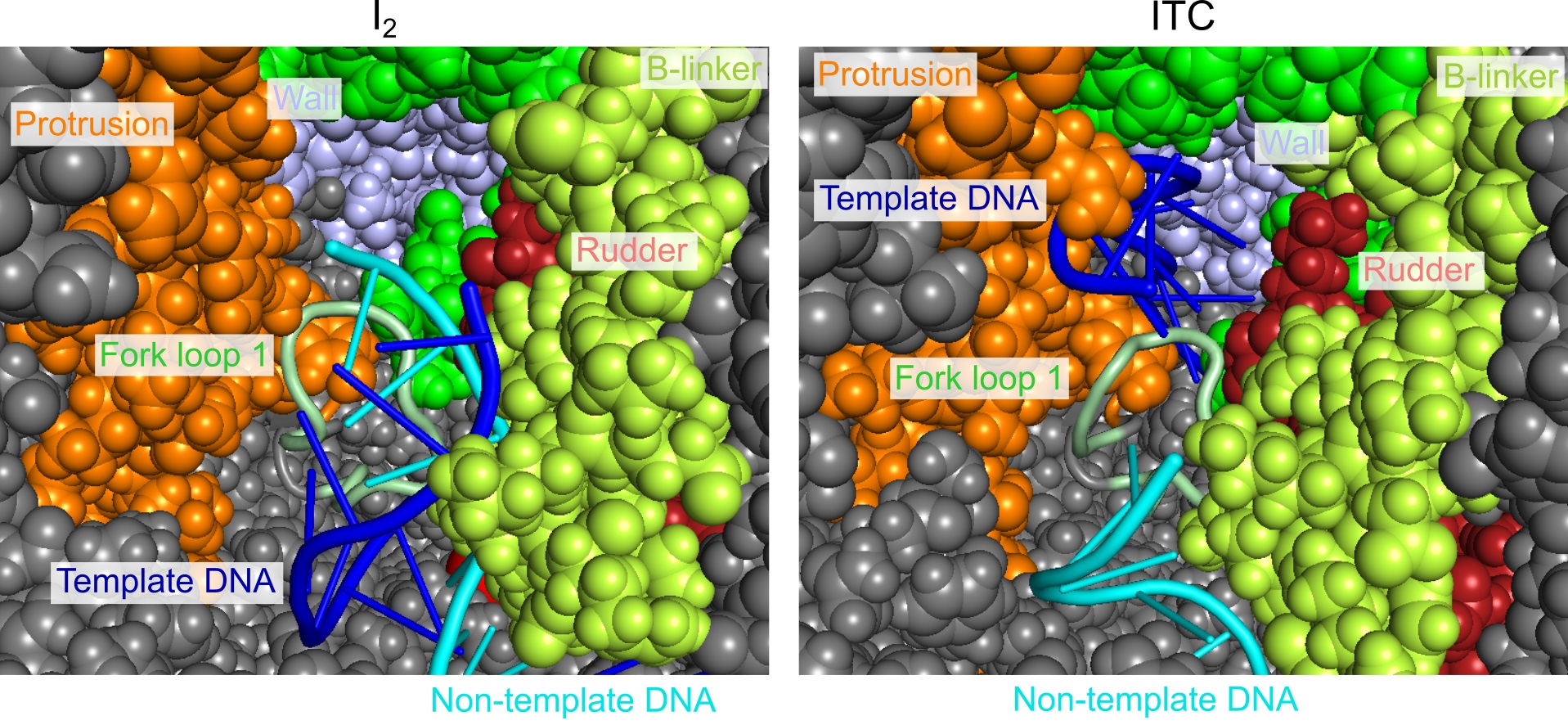

Supplement: Supplementary file 3 [file Image4.JPEG]

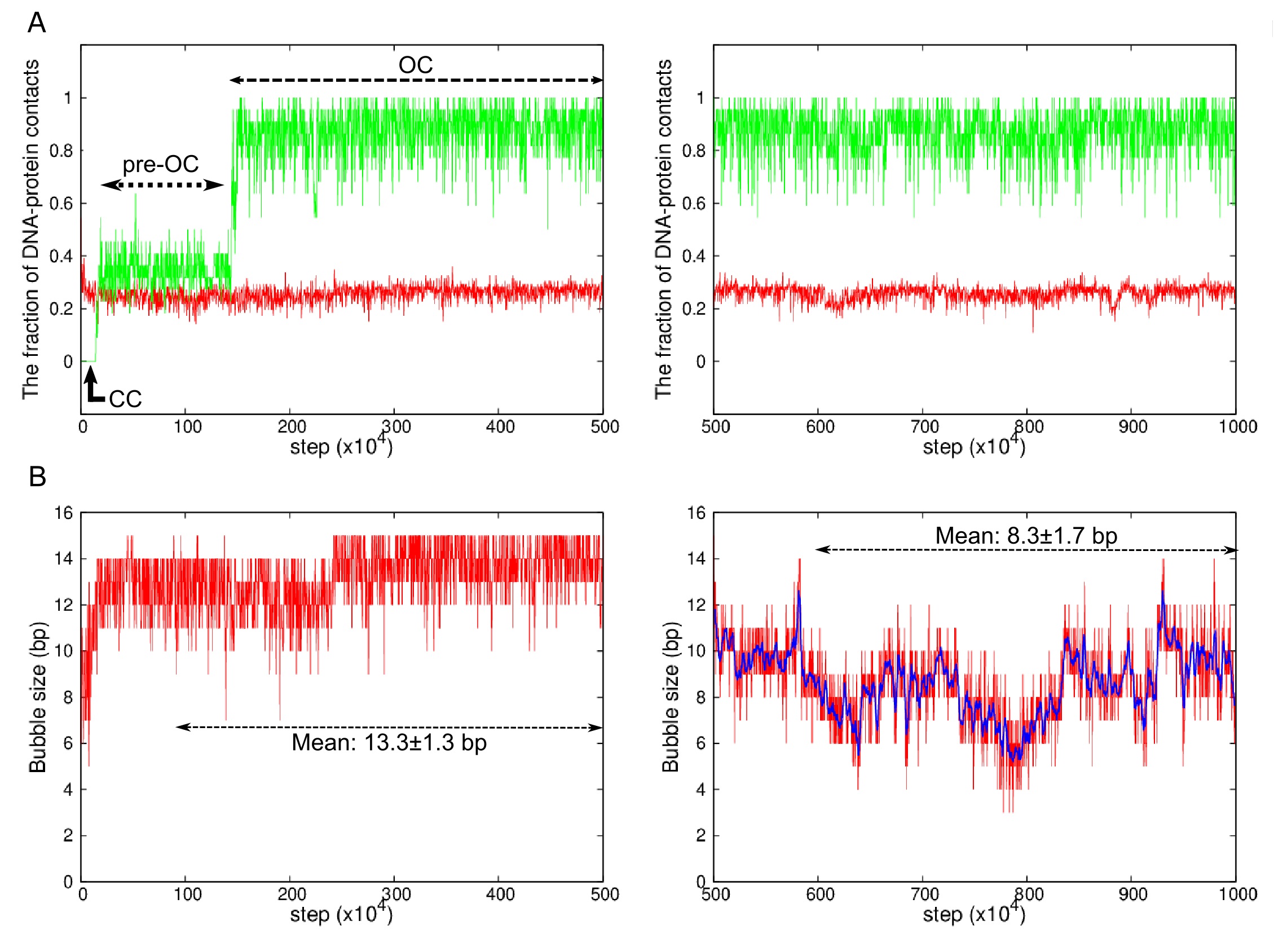

Supplement: Supplementary file 4 [file Image2.JPEG]

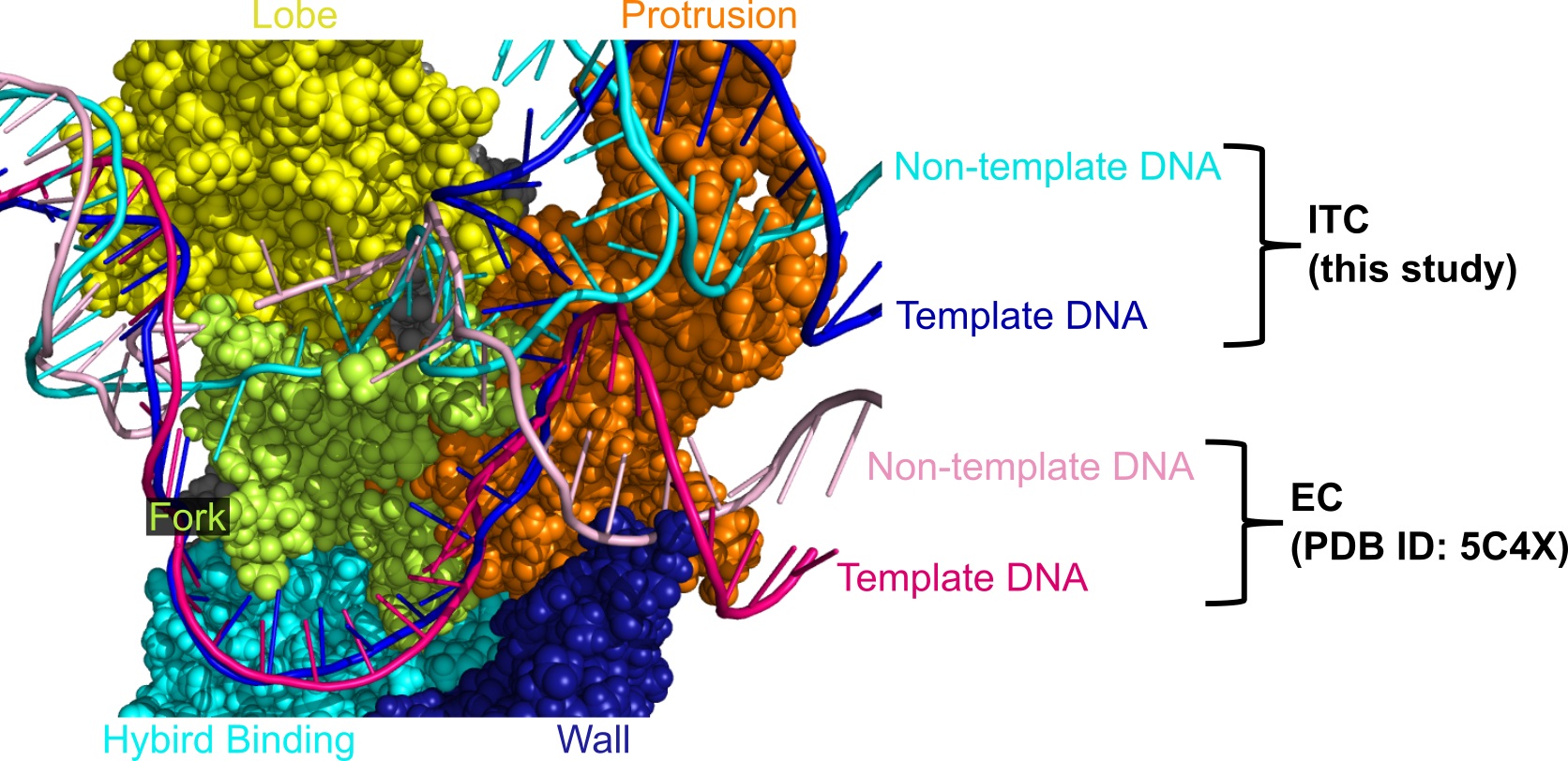

Supplement: Supplementary file 5 [file Image5.JPEG]

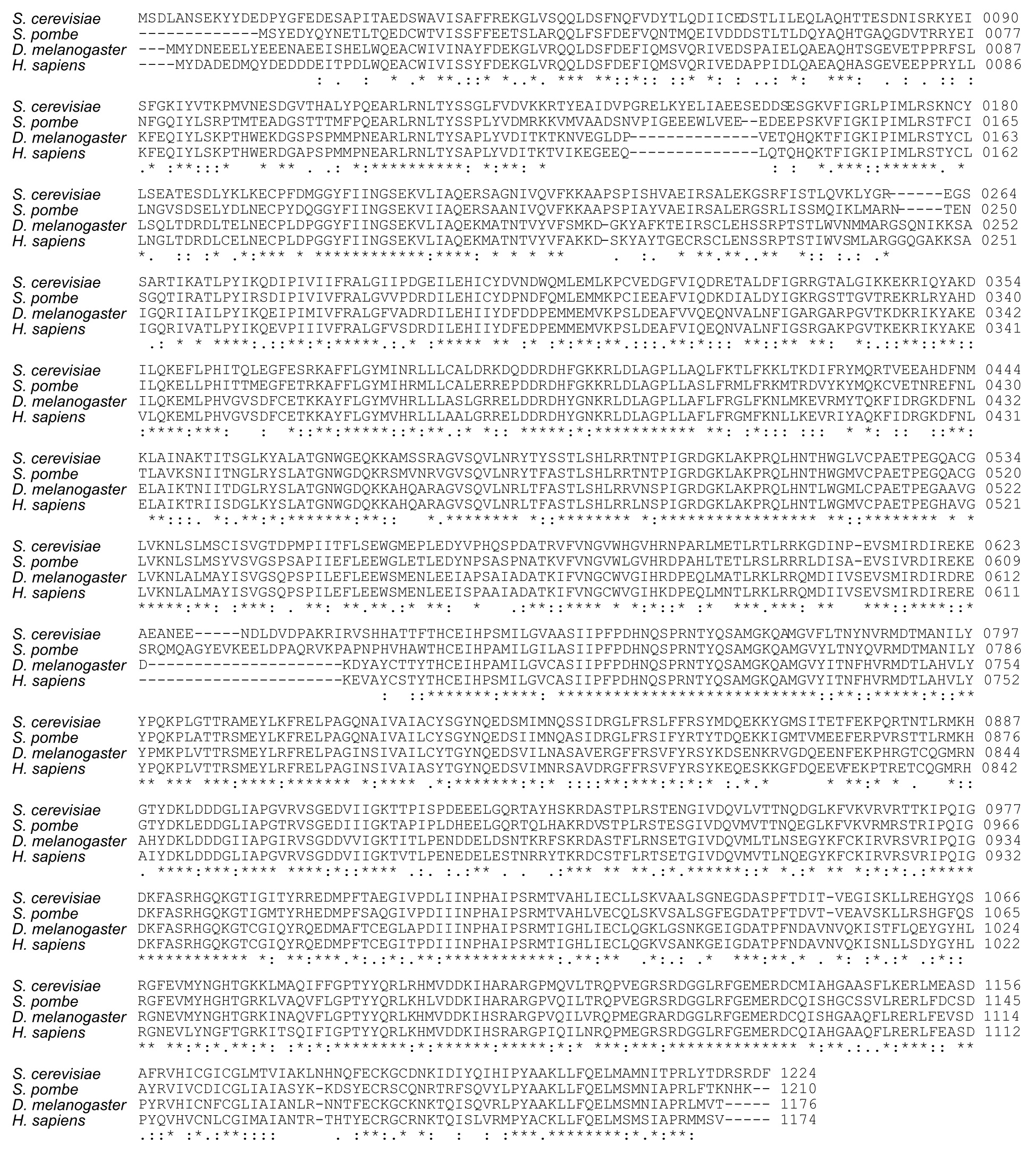

Supplement: Supplementary file 9 [file Image6.JPEG]
