# Supplementary material for: Modeling DNA Opening in the Eukaryotic Transcription Initiation Complexes via Coarse-Grained Models
Source: Front Mol Biosci. 2021 Nov 15;8:772486. doi: 10.3389/fmolb.2021.772486 (PMC8636136; doi:10.3389/fmolb.2021.772486)
Supplement: Supplementary file 8 [file DataSheet1.DOCX]

Supplementary Material

# Energy Function of Protein and DNA

In this study, we used the AICG2+ model for proteins, the 3SPN.2 model for DNA [W. Li et al., 2014; D. M. Hinckley et al., 2013].

The energy function of AICG2+ is given by

$$\boldsymbol{V}_{\boldsymbol{AICG}\boldsymbol{2+}}\left( \boldsymbol{R} | \boldsymbol{R}_{\boldsymbol{0}} \right)\boldsymbol{=}\sum_{\boldsymbol{ibd}} \boldsymbol{K}_{\boldsymbol{b, ibd}}\left( \boldsymbol{b}_{\boldsymbol{ibd}}\boldsymbol{-}\boldsymbol{b}_{\boldsymbol{ibd,0}} \right)^{\boldsymbol{2}}\boldsymbol{+}\boldsymbol{V}_{\boldsymbol{loc}}^{\boldsymbol{flp}}\boldsymbol{+}\sum_{\boldsymbol{j=i+2}} \boldsymbol{\epsilon}_{\boldsymbol{loc,ij}}\mathbf{exp}\left( \boldsymbol{-}\frac{\left( \boldsymbol{r}_{\boldsymbol{ij}}\boldsymbol{-}\boldsymbol{r}_{\boldsymbol{ij}\boldsymbol{0}} \right)^{\boldsymbol{2}}}{\boldsymbol{2}\boldsymbol{W}_{\boldsymbol{ij}}^{\boldsymbol{2}}} \right)\boldsymbol{+}\sum_{\boldsymbol{j=i+3}} \boldsymbol{\epsilon}_{\boldsymbol{loc,ij}}\mathbf{exp}\left( \boldsymbol{-}\frac{\left( \boldsymbol{\phi}_{\boldsymbol{ij}}\boldsymbol{-}\boldsymbol{\phi}_{\boldsymbol{ij}\boldsymbol{0}} \right)^{\boldsymbol{2}}}{\boldsymbol{2}\boldsymbol{W}_{\boldsymbol{\phi,ij}}^{\boldsymbol{2}}} \right)\boldsymbol{+}\sum_{\boldsymbol{i>j+3}}^{\boldsymbol{nat contact}} \boldsymbol{\epsilon}_{\boldsymbol{go,ij}}\left[ \boldsymbol{5}\left( \frac{\boldsymbol{r}_{\boldsymbol{ij}\boldsymbol{0}}}{\boldsymbol{r}_{\boldsymbol{ij}}} \right)^{\boldsymbol{12}}\boldsymbol{-6}\left( \frac{\boldsymbol{r}_{\boldsymbol{ij}\boldsymbol{0}}}{\boldsymbol{r}_{\boldsymbol{ij}}} \right)^{\boldsymbol{10}} \right]\boldsymbol{+}\sum_{\boldsymbol{i>j+3}}^{\boldsymbol{non-native}} \boldsymbol{\epsilon}_{\boldsymbol{ev}}\left( \frac{\boldsymbol{d}}{\boldsymbol{r}_{\boldsymbol{ij}}} \right)^{\boldsymbol{12}}$$

where $\boldsymbol{R}$ and $\boldsymbol{R}_{\mathbf{0}}$ is Cartesian coordinates of the Cα atoms of proteins under simulations and the corresponding coordinates at the native structures. Similarly, all the variables with the subscript 0 mean the parameters of which values take the those of the corresponding variables at the native structure. Each term is summarized below.

- 1^st^ term: Energy related to the virtual bond length. $K_{b,ibd}$ is the parameter, $b_{ibd}$ is the $ibd$-th virtual bond length defined $|\boldsymbol{r}_{ibd+1}-\boldsymbol{r}_{ibd}|$ of the simulated protein, where $\boldsymbol{r}_{ibd}$ is the Cartesian coordinate of the $ibd$-th amino acid.
- 2^nd^ term: A generic flexible local potential for the virtual bond angles and dihedral angles [T. Terakawa and S. Takada, 2011]. This potential is a statistical potential derived by the Boltzmann inversion, further followed by the iterative Boltzmann inversion procedure.
- 3^rd^ term:The interaction energy acting between the $i$-th particle and the $i+2$-th particle, where ϵ_(loc,ij) $\epsilon_{loc,ij}$is the depth parameter of the potential, $W_{ij}$is the width parameter of the potential, and $r_{ij}$ is the distance between the $i$-th and $j$-th particles.
- 4^th^ term: The structure-based interaction energy acting between the $i$-th particle and the $i+3$-th particle, where $W_{\phi,ij}$ is the width parameter of the potential and $\phi_{ij}$is the dihedral angle formed by the four particles from the $i$-th to the $i+3$-th.
- 5^th^ term: The structure-based contact energy (the so-called Go potential) acting on a particle pair with native contacts, where $\epsilon_{go,ij}$is the depth parameter of the potential.
- 6^th^ term: The energy acting on a particle pair that has no native contacts, exhibiting excluded volume effect. $\epsilon_{ev}$ is a coefficient and $d$ is the radius.

The energy function of 3SPN.2 is given by

$$\boldsymbol{V}_{\boldsymbol{3}\boldsymbol{PN.2}}\boldsymbol{=}\boldsymbol{V}_{\boldsymbol{bond}}^{\boldsymbol{3}\boldsymbol{SPN.2}}\boldsymbol{+}\boldsymbol{V}_{\boldsymbol{angle}}^{\boldsymbol{3}\boldsymbol{SPN.2}}\boldsymbol{+}\boldsymbol{V}_{\boldsymbol{dihedral}}^{\boldsymbol{3}\boldsymbol{SPN.2}}\boldsymbol{+}\boldsymbol{V}_{\boldsymbol{b-stack}}^{\boldsymbol{3}\boldsymbol{SPN.2}}\boldsymbol{+}\boldsymbol{V}_{\boldsymbol{bp}}^{\boldsymbol{3}\boldsymbol{SPN.2}}\boldsymbol{+}\boldsymbol{V}_{\boldsymbol{c-stack}}^{\boldsymbol{3}\boldsymbol{SPN.2}}\boldsymbol{+}\boldsymbol{V}_{\boldsymbol{excluded}}^{\boldsymbol{DNA}}\boldsymbol{+}\boldsymbol{V}_{\boldsymbol{ele}}$$

Where each term is summarized below.

- 1^st^ term: Energy related to bond stretching.

$$\boldsymbol{V}_{\boldsymbol{bond}}^{\boldsymbol{3}\boldsymbol{SPN.2}}\mathbf{=}\sum_{\boldsymbol{i}} \left[ \boldsymbol{k}_{\boldsymbol{1}}^{\boldsymbol{3}\boldsymbol{SPN.2}}\left( \boldsymbol{r}^{\boldsymbol{i}}\boldsymbol{-}\boldsymbol{r}_{\boldsymbol{0}}^{\boldsymbol{i}} \right)^{\boldsymbol{2}}\boldsymbol{+100}\boldsymbol{k}_{\boldsymbol{1}}^{\boldsymbol{3}\boldsymbol{SPN.2}}\left( \boldsymbol{r}^{\boldsymbol{i}}\boldsymbol{-}\boldsymbol{r}_{\boldsymbol{0}}^{\boldsymbol{i}} \right)^{\boldsymbol{4}} \right]$$

$k_{1}^{3SPN.2}=0.1839$ is a constant, $r^{i}$ is the $i$-th bond length, and the subscript 0 indicates the value in the B-type double-stranded DNA structure.

- 2^nd^ term: Energy related to the bond angle.

$$\boldsymbol{V}_{\boldsymbol{angle}}^{\boldsymbol{3}\boldsymbol{SPN.2}}\boldsymbol{=}\sum_{\boldsymbol{i}} \boldsymbol{k}_{\boldsymbol{a}}^{\boldsymbol{3}\boldsymbol{SPN.2}}\left( \boldsymbol{\theta}^{\boldsymbol{i}}\boldsymbol{-}\boldsymbol{\theta}_{\boldsymbol{0}}^{\boldsymbol{i}} \right)^{\boldsymbol{2}}$$

$k_{a}^{3SPN.2}=128.73$ is a constant and $\theta^{i}$is the $i$-th bond angle.

- 3^rd^ term: Energy related to dihedral angles.

$$\boldsymbol{V}_{\boldsymbol{dihedral}}^{\boldsymbol{3}\boldsymbol{SPN.2}}\boldsymbol{=}\sum_{\boldsymbol{i}} \boldsymbol{-}\boldsymbol{k}_{\boldsymbol{\phi}}^{\boldsymbol{3}\boldsymbol{SPN.2}}\mathbf{exp}\left( \boldsymbol{-}\frac{\left( \boldsymbol{\phi}^{\boldsymbol{i}}\boldsymbol{-}\boldsymbol{\phi}_{\boldsymbol{0}}^{\boldsymbol{i}} \right)^{\boldsymbol{2}}}{\boldsymbol{2}\boldsymbol{\sigma}_{\boldsymbol{\phi i}}^{\boldsymbol{2}}} \right)$$

$k_{\phi}^{3SPN.2}=5.1492$ is a constant, $\phi^{i}$ is the $i$-th dihedral angle, and $\sigma_{\phi i}$ is the width parameter of the potential.

- 4^th^, 5^th^, and 6^th^ terms: Energy related to base interaction. These three terms have distance-dependent and orientation-dependent characteristics. The distance dependence is expressed by the Morse potential, which is divided into the following repulsion and attraction.

$$\boldsymbol{U}_{\boldsymbol{m}}^{\boldsymbol{rep}}\left( \boldsymbol{r}_{\boldsymbol{ij}} \right)\boldsymbol{=}\left\{ \begin{aligned} \boldsymbol{\epsilon}_{\boldsymbol{ij}}\left[ \boldsymbol{1-}\mathbf{exp}\left( \boldsymbol{-}\boldsymbol{\alpha}_{\boldsymbol{ij}}\left( \boldsymbol{r}_{\boldsymbol{ij}}\boldsymbol{-}\boldsymbol{r}_{\boldsymbol{0,ij}} \right) \right) \right]^{\boldsymbol{2}} \boldsymbol{(}\mathbf{where} \boldsymbol{r}_{\boldsymbol{ij}}\boldsymbol{<}\boldsymbol{r}_{\boldsymbol{0,ij}}\boldsymbol{)} \\ \boldsymbol{0} \mathbf{(where}\boldsymbol{r}_{\boldsymbol{ij}}\boldsymbol{\geq}\boldsymbol{r}_{\boldsymbol{0,ij}}\mathbf{)} \end{aligned} \right.$$

$$\boldsymbol{U}_{\boldsymbol{m}}^{\boldsymbol{attr}}\left( \boldsymbol{r}_{\boldsymbol{ij}} \right)\boldsymbol{=}\left\{ \begin{aligned} \boldsymbol{-}\boldsymbol{\epsilon}_{\boldsymbol{ij}} \boldsymbol{(}\mathbf{where} \boldsymbol{r}_{\boldsymbol{ij}}\boldsymbol{<}\boldsymbol{r}_{\boldsymbol{0,ij}}\boldsymbol{)} \\ \boldsymbol{\epsilon}_{\boldsymbol{ij}}\left[ \boldsymbol{1-}\mathbf{exp}\left( \boldsymbol{-}\boldsymbol{\alpha}_{\boldsymbol{ij}}\left( \boldsymbol{r}_{\boldsymbol{ij}}\boldsymbol{-}\boldsymbol{r}_{\boldsymbol{0,ij}} \right) \right) \right]^{\boldsymbol{2}} \boldsymbol{(}\mathbf{where}\boldsymbol{r}_{\boldsymbol{ij}}\boldsymbol{\geq}\boldsymbol{r}_{\boldsymbol{0,ij}}\boldsymbol{)} \end{aligned} \right.$$

$\epsilon_{ij}$ is the depth parameter of the potential and $\alpha_{ij}$is the width parameter of the potential. The gravitational term also has an orientation dependence and is given by

$$\boldsymbol{f}\left( \boldsymbol{K,}\boldsymbol{\Delta}\boldsymbol{\theta} \right)\boldsymbol{=}\left\{ \begin{aligned} \boldsymbol{1} \left( \boldsymbol{-}\frac{\boldsymbol{\pi}}{\boldsymbol{2}\boldsymbol{K}}\boldsymbol{<}\boldsymbol{\Delta}\boldsymbol{\theta<}\frac{\boldsymbol{\pi}}{\boldsymbol{2}\boldsymbol{K}}\mathbf{のとき} \right) \\ \boldsymbol{1-}\mathbf{cos}^{\mathbf{2}} \left( \boldsymbol{K}\boldsymbol{\Delta}\boldsymbol{\theta} \right) \left( \boldsymbol{-}\frac{\boldsymbol{\pi}}{\boldsymbol{K}}\boldsymbol{<}\boldsymbol{\Delta}\boldsymbol{\theta<-}\frac{\boldsymbol{\pi}}{\boldsymbol{2}\boldsymbol{K}}\mathbf{または}\frac{\boldsymbol{\pi}}{\boldsymbol{2}\boldsymbol{K}}\boldsymbol{<}\boldsymbol{\Delta}\boldsymbol{\theta<}\frac{\boldsymbol{\pi}}{\boldsymbol{K}}\mathbf{のとき} \right) \\ \boldsymbol{0} \left( \boldsymbol{\Delta}\boldsymbol{\theta<-}\frac{\boldsymbol{\pi}}{\boldsymbol{K}}\mathbf{または}\frac{\boldsymbol{\pi}}{\boldsymbol{K}}\boldsymbol{<}\boldsymbol{\Delta}\boldsymbol{\theta}\mathbf{のとき} \right) \end{aligned} \right.$$

where $\Delta\theta=\theta-\theta_{0}$ is the angle deviation and $K$ is the range width of the angle.

The 4^th^ term is the stacking energy between neighboring bases on a single strand.

$$\boldsymbol{V}_{\boldsymbol{b-stack}}^{\boldsymbol{3}\boldsymbol{SPN.2}}\boldsymbol{=}\sum\left\{ \begin{aligned} \boldsymbol{U}_{\boldsymbol{m}}^{\boldsymbol{rep}}\left( \boldsymbol{r}_{\boldsymbol{ij}} \right)\boldsymbol{+f}\left( \boldsymbol{\Delta}\boldsymbol{\theta}_{\boldsymbol{BS,ij}} \right)\boldsymbol{U}_{\boldsymbol{m}}^{\boldsymbol{attr}}\left( \boldsymbol{r}_{\boldsymbol{ij}} \right) \boldsymbol{(}\mathbf{where} \boldsymbol{r}_{\boldsymbol{ij}}\boldsymbol{<}\boldsymbol{r}_{\boldsymbol{0,ij}}\boldsymbol{)} \\ \boldsymbol{f}\left( \boldsymbol{\Delta}\boldsymbol{\theta}_{\boldsymbol{BS,ij}} \right)\boldsymbol{U}_{\boldsymbol{m}}^{\boldsymbol{attr}}\left( \boldsymbol{r}_{\boldsymbol{ij}} \right) \boldsymbol{(}\boldsymbol{where r}_{\boldsymbol{ij}}\boldsymbol{\geq}\boldsymbol{r}_{\boldsymbol{0,ij}}\boldsymbol{)} \end{aligned} \right.$$

Here, $r_{ij}$ is the distance between two neighboring bases on a single strand, and $\theta_{BS}$ is the angle between the vector connecting a base to a sugar of a nucleotide and the vector connecting the same base to one of its neighbors.

The 5^th^ term is the interaction energy (base pair energy) for base pairs of double-stranded DNA that are connected through hydrogen bonds.

$$\boldsymbol{V}_{\boldsymbol{bp}}^{\boldsymbol{3}\boldsymbol{SPN.2}}\boldsymbol{=}\sum\left\{ \begin{aligned} \boldsymbol{U}_{\boldsymbol{m}}^{\boldsymbol{rep}}\left( \boldsymbol{r}_{\boldsymbol{ij}} \right)\boldsymbol{+0.5}\left( \boldsymbol{1+}\cos\boldsymbol{\Delta}\boldsymbol{\phi}_{\boldsymbol{1}} \right)\boldsymbol{f}\left( \boldsymbol{\Delta}\boldsymbol{\theta}_{\boldsymbol{1,ij}} \right)\boldsymbol{f}\left( \boldsymbol{\Delta}\boldsymbol{\theta}_{\boldsymbol{2,ij}} \right)\boldsymbol{U}_{\boldsymbol{m}}^{\boldsymbol{attr}}\left( \boldsymbol{r}_{\boldsymbol{ij}} \right) \boldsymbol{(}\boldsymbol{where r}_{\boldsymbol{ij}}\boldsymbol{<}\boldsymbol{r}_{\boldsymbol{0,ij}}\boldsymbol{)} \\ \boldsymbol{0.5}\left( \boldsymbol{1+}\cos\boldsymbol{\Delta}\boldsymbol{\phi}_{\boldsymbol{1}} \right)\boldsymbol{f}\left( \boldsymbol{\Delta}\boldsymbol{\theta}_{\boldsymbol{1,ij}} \right)\boldsymbol{f}\left( \boldsymbol{\Delta}\boldsymbol{\theta}_{\boldsymbol{2,ij}} \right)\boldsymbol{U}_{\boldsymbol{m}}^{\boldsymbol{attr}}\left( \boldsymbol{r}_{\boldsymbol{ij}} \right) \boldsymbol{(}\boldsymbol{where r}_{\boldsymbol{ij}}\boldsymbol{\geq}\boldsymbol{r}_{\boldsymbol{0,ij}}\boldsymbol{)} \end{aligned} \right.$$

where $r_{ij}$ is the distance between the two bases connected by hydrogen bonds, $\theta_{1}$ is the angle formed by the vector connecting the sugar and base in the $i$-th nucleotide to the vector connecting the same $i$-th base to the $j$-th base hydrogen bonded to it, $\theta_{2}$ is the angle formed by the vector connecting the sugar and base in the $j$-th nucleotide to the vector connecting the same $j$-th base to the $i$-th base hydrogen bonded to it, and $\phi_{1}$is the dihedral angle formed by the four particles of the $i, j$-th sugar and base.

The 6^th^ term is the interaction energy (cross-stacking energy) between the $i$-th base on a single strand and the $j$-th base next to it that is hydrogen bonded to it.

$$\boldsymbol{V}_{\boldsymbol{c-stack}}^{\boldsymbol{3}\boldsymbol{SPN.2}}\mathbf{=}\sum\boldsymbol{f}\left( \boldsymbol{\Delta}\boldsymbol{\theta}_{\boldsymbol{3,ij}} \right)\boldsymbol{f}\left( \boldsymbol{\Delta}\boldsymbol{\theta}_{\boldsymbol{CS,ij}} \right)\boldsymbol{U}_{\boldsymbol{m}}^{\boldsymbol{attr}}\boldsymbol{(}\boldsymbol{r}_{\boldsymbol{ij}}\boldsymbol{)}$$

where $r_{ij}$ is the distance between the two nucleotides of the interest, $\theta_{3}$ is the angle formed by the vector connecting the sugar and base in the $i$-th nucleotide and the same vector in the nucleotide hydrogen-bonded to the $i$-th base (not the $j$-th nucleotide), and $\theta_{CS}$ is the angle formed by the vector connecting the sugar and base in the $i$-th nucleotide $\theta_{CS}$ is the angle formed by the vector connecting the sugar and the base in the $i$-th nucleotide and the vector connecting the $i$-th base and the $j$-th base.

- 7^th^ term: Energy from excluded volume effect.

$$\boldsymbol{V}_{\boldsymbol{excluded}}^{\boldsymbol{3}\boldsymbol{SPN.2}}\boldsymbol{=}\sum_{\boldsymbol{i<j}} \left\{ \begin{aligned} \boldsymbol{\epsilon}_{\boldsymbol{r}}\left[ \left( \frac{\boldsymbol{\sigma}_{\boldsymbol{ij}}}{\boldsymbol{r}_{\boldsymbol{ij}}} \right)^{\boldsymbol{12}}\boldsymbol{-2}\left( \frac{\boldsymbol{\sigma}_{\boldsymbol{ij}}}{\boldsymbol{r}_{\boldsymbol{ij}}} \right)^{\boldsymbol{6}} \right]\boldsymbol{+}\boldsymbol{\epsilon}_{\boldsymbol{r}} \boldsymbol{(}\boldsymbol{r}_{\boldsymbol{ij}}\boldsymbol{<}\boldsymbol{\sigma}_{\boldsymbol{ij}}\mathbf{のとき}\boldsymbol{)} \\ \boldsymbol{0} \boldsymbol{(}\boldsymbol{r}_{\boldsymbol{ij}}\boldsymbol{\geq}\boldsymbol{\sigma}_{\boldsymbol{ij}}\mathbf{のとき}\boldsymbol{)} \end{aligned} \right.$$

$\epsilon_{r}$ is the depth parameter of the potential, $\sigma_{ij}$ is the particle radius, and $r_{ij}$ is the distance between the $i$-th particle and the $j$-th particle.

- 8^th^ term: Energy from electrostatic interactions.

$$\boldsymbol{V}_{\boldsymbol{ele}}\boldsymbol{=}\sum_{\boldsymbol{i<j}}^{\boldsymbol{N}} \frac{\boldsymbol{q}_{\boldsymbol{i}}\boldsymbol{q}_{\boldsymbol{j}}}{\boldsymbol{4}\boldsymbol{\pi}\boldsymbol{\epsilon}_{\boldsymbol{0}}\boldsymbol{\epsilon}_{\boldsymbol{k}}\boldsymbol{r}_{\boldsymbol{ij}}}\mathbf{exp}\left( \boldsymbol{-}\frac{\boldsymbol{r}_{\boldsymbol{ij}}}{\boldsymbol{\lambda}_{\boldsymbol{D}}} \right)\mathbf{,} \boldsymbol{\lambda}_{\boldsymbol{D}}\boldsymbol{=}\left( \frac{\boldsymbol{\epsilon}_{\boldsymbol{0}}\boldsymbol{\epsilon}_{\boldsymbol{k}}\boldsymbol{k}_{\boldsymbol{B}}\boldsymbol{T}}{\boldsymbol{2}\boldsymbol{N}_{\boldsymbol{A}}\boldsymbol{e}^{\boldsymbol{2}}\boldsymbol{I}} \right)^{\boldsymbol{0.5}}$$

$q_{i}$ is the charge, $\epsilon_{0}$ is the electrical constant, $\epsilon_{k}$ is the relative permittivity, $\lambda_{D}$ is the Debye length, $N_{A}$is the Avogadro number, and $I$ is the ionic strength (salinity). The ionic strength $I$ is defined as follows ($c_{i}$ is the molar density).

$$\boldsymbol{I}\mathbf{=0.5}\sum\boldsymbol{z}_{\boldsymbol{i}}^{\boldsymbol{2}}\boldsymbol{c}_{\boldsymbol{i}}\mathbf{,} \boldsymbol{z}_{\boldsymbol{i}}\boldsymbol{=}\frac{\boldsymbol{q}_{\boldsymbol{i}}}{\boldsymbol{e}}$$

The energy from electrostatic interactions also works between proteins and amino acids. The value of charge $q_{i}$ is set to +1 for arginine (Arg) and lysine (Lys), -1 for aspartic acid (Asp) and glutamic acid (Glu), 0 for other amino acids, and -0.6 for phosphoric acid in DNA when phosphoric acid repels each other (considering counterion condensation), and -1.0 when it interacts with amino acids by attraction. The phosphoric acid in DNA is set to -0.6 when the phosphoric acid repels each other (considering counterion condensation) and -1.0 when it interacts with amino acids by attraction.

Between the protein and DNA, we employed the Go potential [S. Takada, 2019] given by

$$\boldsymbol{\epsilon}_{\boldsymbol{go}}\left[ \boldsymbol{5}\left( \frac{\boldsymbol{r}_{\boldsymbol{ij}\boldsymbol{0}}}{\boldsymbol{r}_{\boldsymbol{ij}}} \right)^{\boldsymbol{12}}\boldsymbol{-6}\left( \frac{\boldsymbol{r}_{\boldsymbol{ij}\boldsymbol{0}}}{\boldsymbol{r}_{\boldsymbol{ij}}} \right)^{\boldsymbol{10}} \right]$$

where $\epsilon_{go}$ is the coefficient and $r_{ij}$ is the distance between the two particles; increasing the value of $\epsilon_{go}$ results in a stronger interaction between the protein and the DNA.

# Supplementary Figures

**Figure S1:** **Coarse-grained MD simulation from the CC state without the mismatch DNA sequence.** Results of a representative trajectory is shown. (A) Superposition of DNAs in the initial (t=0, yellow) and final (t=500 x 10^4^ MD steps, orange) states. Black dashed square indicates the distorted region of the promotor. (B) The time course of the fractions of protein-DNA contacts specific to CC (red) and OC (green). (C) The time course of the DNA bubble size.

**Figure S2: Coarse-grained MD simulation for the transition from the CC to OC states (this trajectory is different from that in Figure 2).** (A) The time course of the fractions of protein-DNA contacts specific to CC (red) and OC (green). (B) The time course of the DNA bubble size. In (A) and (B), the left (right) panels are from the first (second) halves of MD simulations with (without) the DNA mismatch. The blue curve in the right panel in (B) shows a moving average over 11 points.

**Figure S3:** **Coarse-grained MD simulation for the transition from the OC to ITC states with a weakened attraction between DNA and the E-wing residues.** Results of typical two trajectories are shown in red and blue curves. (A) The time course of the fractions of protein-DNA contacts specific to ITC states. (B) The time course of the DNA bubble size. (C) The time courses of the distance between the centers of mass of the fork loop 1 of the Pol II Rpb2 (468-476 residues) and the B-linker in TFIIB (99-102 residues). Green dashed lines, a characteristic distance for the template DNA to pass through the fork loop 1.

**Figure S4:** **Close-up views around the fork loop 1 (pale green) of the Rpb2 in the I_2_ (left) and ITC (right) states.** In the I_2_ state, both template and non-template DNA strands are in the near side of fork loop 1, whereas, in the ITC state, template and non-template DNA strands are in the near-side and the other side of the fork loop 1.

**Figure S5: Comparison of the DNA bubbles in ITC (blue and cyan) and in the elongation complex (EC; PDB ID 5C4X) (magenta and pink).** The DNA bubble structures in the two cases are similar.

**Figure S6:** **Multiple sequence alignment of Rpb2.** "*", identity; ":", strong similarity; ".", weak similarity.

**Movie S1: Coarse-grained MD simulation for the transition from the CC to OC states.**

**Movie S2: Coarse-grained MD simulation for the transition from the OC to ITC states.**

# Reference

Go, N. (1983). Theoretical studies of protein folding. Annual review of biophysics and bioengineering.

Hinckley, D. M., Freeman, G. S., Whitmer, J. K., de Pablo, J. J. (2013). An experimentally-informed coarse-grained 3-site-per-nucleotide model of DNA: Structure, thermodynamics, and dynamics of hybridization. J. Chem. Phys., 139, 144903.

Li, W., Wang, W., Takada, S. (2014). Energy landscape views for interplays among folding, binding, and allosteyof calmodulin domains. Proc. Natl. Acad. Sci., 108 (9), 3504.

Takada, S. (2019). Gō model revisited. Biophys. Physicobiol., 16.

Terakawa, T., Takada, S. (2011). Multiscale Ensemble Modeling of Intrinsically Disordered Proteins: p53 N-Terminal Domain. Biophys. J., 101, 1450–1458.
